# Observations on Congenital Fissure of the Palate, with Some Remarks on Articulation and Impediments of Speech

**Published:** 1847-06

**Authors:** Charles W. Stearns

**Affiliations:** Surgeon, London.


### ARTICLE V.

*Observations on Congenital Fissure of the Palate, with some Remarks on Articulation and Impediments of Speech.* By CHARLES W. STEARNS, Esq., Surgeon, London.

PHYSIOLOGISTS will be found not to have devoted much space to describing in detail the *modus operandi* of the vocal organs in performing the functions of articulation. Perhaps the particular method of severally employing the lips, the tongue, and the palate, or the shape required to be given to the mouth in order to produce a given sound, is thought so simple a matter that every one can as well observe for themselves. But that they fail to do so, and even find it difficult, is proved by the attention necessary to acquire any one of the sounds peculiar to a foreign language. Therefore it happens that the *ä*, *kn*, and *ich* of the German; the *u* and *oi* of the French, and the *th* of the English, remain the shibboleth of all strangers. This is wholly the consequence of habit, inasmuch as the same physical organization, the same vocal apparatus, is common to all races and nations: this apparatus is capable of producing but a certain number of distinct sounds, and each of these sounds can be produced in only one way.* It is the habitual predominance of certain of these sounds, and the characteristic grouping of certain consonants, that constitutes the distinguishing features of a language. Hence, philologists have been obliged to consider the subject of articulation and voice in connection with their pursuits, and except in their dictionaries and grammars we shall find little elsewhere relating to it. Fortunately for our purpose, we can refer to Dr. Arnott's "Elements of Physics," where, under the head of "Voice and Speech," a complete analytical view is presented. From this we had designed to make some extracts, as furnishing the only satisfactory exposition with which we were acquainted, but the book itself is so widely circulated, and each paragraph of the portion to which reference is here made is so important to a complete understanding of the whole, and so closely connected with it, that we must content ourselves with only copying the author's tabular arrangement of the articulations:—

| Labial. | Palatal. | Guttural. | | |
|---|---|---|---|---|
| P† | T L | K | . . | Mute |
| B† | D | G | . . | Semi-mute |
| M | N | *ng* | . (*on*) | Semi-vocal or nasal |
| F† | *th* S *sh* | *ch* | . (H) | Aspirate |
| V† | *th* Z J | *gh* | . . | Vocal aspirate |
| P*r* | R | *ghr* | . . | Vibratory |

The above parallelogram represents the cavity of the mouth, considered as a tube or hollow cylinder, having three principal divisions—one on the left, representing the region of the *lips;* that on the right, the region of the *throat;* that in the middle, the region of the *palate.* These regions have been styled by Dr. Arnott, "the three oral positions," each of which furnishes a certain number of sounds or articulations peculiar to itself, as

* This fact has suggested the idea of an *universal* language. It has also been made the basis of systems of short-hand writing; besides the repeated attempt to develop some practical plan of *mnemonics,* or artificial memory.

† By comparing these articulations, we at once discover one cause (a *physical* cause) of the gradual corruption of language. Thus: *amavi* was once *amafui,* and afterwards *amavui,* &c. In like manner, *bitumen* stands for *pitumen,* from *pituita.* Also *fera,* from the Greek *ther,* and *ambo,* from *ampho,* &c.

indicated by the letters. These sounds or articulations are produced by interrupting the current of voice at any one of these "oral positions," and there being several ways of making this interruption in each of these positions, we obtain, therefore, a multiplied number of sounds. Thus, if the tube or cylinder of the mouth be left open, so that the sound, passing outwards, meets with no interruption until it arrives at the lips, which being simply closed so as to obstruct its final exit, we then have the letter P, which is therefore termed a *labial mute*. But if this obstruction is attended by a momentary accumulation of sound in the cavity behind the organ making the closure, so as to cause a slight distention, that would give a half-mute, as B instead of P. There is a similar difference between T and D, and also between K and G, as between P and B. In T and D, the necessary closure is made by bringing the edge of the tongue in contact with the gums of the upper teeth throughout its whole circumference. In K, the closure is made by carrying the root of the tongue against the soft palate, thus obstructing the passage of the voice almost at its starting point. The letters just mentioned, (which are exhibited in Dr. Arnott's table as mutes and semi-mutes,) being the most important in illustration of our subject, it is not necessary to speak of the others.

Everything, then, seems to depend upon this interruption or closure, which is wholly a mechanical function.* It is easy, therefore, to see how such a defect in a part of the mechanism as that of fissure of the palate, by preventing this closure or occlusion, renders articulation of some of the letters impossible and vitiates the character of all the others.†

* In proof of this, see how perfectly the sound of G-hard can be imitated by taking a bottle of water, and inverting it. Hence, no doubt, the expressive words, *guggle* and *gurgle*, which perhaps were used in the infancy of language, and led afterwards to the adoption of the letter *g* (a simple abstraction) in the formation of other words; so *z* from buzz.

† It may be said, that if so much really depends upon the occlusion of sound, then merely pinching together the nostrils ought to afford some diminution of the impediment. But that would be making a permanent closure, and at a point far removed, besides permitting the vibrations of sound to mingle with a considerable body of air contained in the nasal sinuses. Moreover, it may be that the soft palate performs one office simi-

The indistinctness of utterance is usually proportioned to the extent of the lesion. Thus, where the fissure extends as far as the alveolar processes, that patient loses several of the letters which another, with only a portion of the soft palate involved, is able to produce with considerable distinctness. But this relative extent of the impediment to that of the fissure is not always exactly maintained. This may happen from one individual possessing a more correct ear than another, which makes him habitually careful to employ what means of articulation he may have to the best advantage.

We have already remarked, that it was difficult for any person to criticise the peculiarities of his own voice, because he has no means of comparison, but persons who themselves fortunately possess all the faculties of speech would imagine that others laboring under a serious impediment must comprehend exactly wherein their own faults consist, and that the impression made by it upon their own sense of hearing must be the same as that received by those around them. They always fail to recognise their characteristic nasal tone, as I had occasion to observe in a particular instance of one young gentleman who had a cultivated musical ear, and could distinguish perfectly well each individual articulation on which he was deficient, but the equally obvious peculiarity of sound did not strike him.

Fissure of the palate, then, does not preclude the utterance of the vowel sounds, but only renders them strongly nasal. This is most conspicuous in the short sounds, as *a* in hat, *e* in met, *i* in tic, &c. as those require an increased expenditure of muscular force, the effects of which, as we before remarked, is to drive more of the sound backwards into the nasal passages.

The articulations which occasion the most serious difficulty are, first, the semi-mutes *g*, *d* and *b*, (placed in the second line lar to that of the tympanum of the ear, modifying the sound impinging one side, by the presence of an unconfined volume of air on the other side. Closing the nostrils, therefore, would impede the regular function in one instance, as completely as accidental obstruction of the Eustachian tube is known to do in the other. The nasal passages serve also to continue the supply of air during the act of speaking, as in the common plan of ventilation.

of Dr. Arnott's table,) and after these are the mutes *k*, *t* and *p*, in the line above. Those in the fourth and fifth lines (though more or less indistinct) are usually perceptible.

Of all the letters, *g* hard (as in gig) is the most difficult, that articulation being wholly unattainable, without the aid of the velum palati, to effect a perfect occlusion; and without this, the more effort made in the attempt, the more manifest the inability appears; the same is also true of the other guttural, *k*. If this letter happens to accompany one of the short vowels, then there is often a sort of counterfeit articulation of it; but if we cause the word to be spoken slowly, we shall discern that the letter is wanting; in cases more limited, it is often present. *D* is found to be the next most difficult, as it is a semi-mute, requiring, therefore, like the *g*, that the occlusion be more complete, and longer continued, than for *k* or *t*. When the fissure includes the roof of the mouth, *d* is the most noticeable of all the defective articulations, and in less marked cases it is apt to be mistaken for *n*, as manner for madder. In one case, that of a boy of thirteen, who had been operated upon for hare-lip, there was always a vicarious attempt to make the necessary interruption for the letter *d*, by thrusting the tongue quite out of the mouth, so as to meet the upper lip.

With regard to the letters classed in the table as *aspirates* and *vocal aspirates*, it is only necessary to remark, that *z* and *j* are the most imperfectly articulated; *z*, as in Zuyder Zee, is much more difficult than its corresponding palatal *s*, in the line above. The sound of *j* very often represented by the soft *g*, as in George, stranger, judge, and the like, is generally very faintly heard, and is one of those deficient letters, the absence of which the ear of the patient fails to detect.

It is not worth while to go through the whole alphabet, as we have noticed those articulations most important in connection with our subject. Our language has one unfortunate peculiarity, in often allowing one letter to stand in place of another, the sound of the first being retained, which is, in effect, calling a letter by a wrong name. Thus, *t* may stand for *sh*, as in nation, and *s* often stands for *z*, as in is, dismal, &c.; while the five letters, *c*, *q*, *x*, *w* and *y*, will be found to have no characteristic sound of their own, but are always substituted for some other letter, which, for no good reason, is temporarily set aside.

The *modus operandi* of articulation is not so readily comprehended by one whose organs are congenitally defective. From always having been accustomed to employ them in a manner peculiar to himself, he is not able at once to change a long established habit, and it will require some teaching to make him understand how to manage the tongue or the lips, in order to produce a particular sound, as that of D, K, or L, and probably all the palatal or guttural letters. In one case of fissure of the palate, the patient confessed that he could not see the necessary connection between the words and syllables and the letters out of which they were formed; and it should not be thought strange, if, being unprovided with a perfect apparatus for producing the usual modifications of sound, he should fail to detect them in their rapid transit towards their destined combinations and groups in the formation of language; indeed, that the choice of certain letters for spelling a word was as arbitrary and conventional a matter as the form of the visible characters used in writing it. Persons with vocal organs anatomically defective, may, in consequence, be prevented from comprehending the process of articulation, for the same reason that one born blind is unable to acquire correct notions of color or distance, the method of grouping and combining the elements of speech, being compared to the art of the painter, by which a few primary colors are mixed and blended so as to produce an infinite variety of tints.

Another less obvious characteristic of congenital fissure of the palate, but one of the first practicable importance, remains to be noticed. Not only is the patient unable to retain the sound in the cavity of the mouth long enough to undergo the coining-like process of articulation, as already explained, but he is equally deprived of the power of accumulating sound in the larynx, which is thereby deprived of its appropriate stimulus of distention necessary to rouse it and all its dependencies to the proper and energetic performance of their functions. This will be demonstrated if the patient attempts to make a buzzing noise, like the forcible and prolonged utterance of the letter Z, or that hissing sound sometimes heard in the attempt to suppress a violent fit of laughter, during which, the glosso-laryngeal region externally assumes an inflated appearance. In the occurrence of the semi-mutes B, D, and G, which are characterized by an accumulation of sound (the vocal murmur) behind the organ making the interruption, a similar distention of the larynx takes place when all the organs are in a normal state. Consequently, we observe, in persons of fine vocal powers, as professional singers and public speakers, that there is a good degree of fulness about that particular region of the neck, which is seen during an effort of the voice to be the seat of a great deal of motion, indicating that all the muscles are actively employed. But an opposite condition of the parts is the consequence of fissure, for then the glosso-laryngeal region presents, comparatively, a sunken and emaciated appearance, indicating want of tone from inactivity. The larynx, the position of which is shown by the pomum adami, is seen not to have its proper ascending and descending motion during the act of speech, and on applying the fingers to the parts, little or no vibration will be felt. Nor can the patient succeed by any voluntary effort in disturbing this long-accustomed passive habit of that organ, because it has never yet felt that stimulus of distention necessary to bring the muscles concerned under the control of the will, by first making their possessor concious of their existence. So fixed is this dormant habit of the larynx and its dependencies, that it is probable, if the palate were to be suddenly restored, by miracle, as it were, to a perfectly normal state, that the original impediment would, in a great degree, remain until use and discipline had served to develope the newly acquired powers.

In these cases, then, we seem to have an exception to that benevolent disposition nature usually manifests to supply a defect of one organ by increased activity of some other; for here the laryngeal portion of the vocal mechanism is, no doubt, entire, yet rendered less efficient by lesion of the palatine portion. It is, moreover, probable, that the tone or positive strength of the muscular tissue forming the substance of the tongue and lips, is also diminished from the same cause. Thus a flaw in a single link of the chain has the effect to impair the usefulness of the whole series and any one who glances at such a case will see at once what it is desirable to do—that this single broken link should be restored, and that a lesion, which is simply and obviously a *mechanical* imperfection, must admit of some remedy within the reach of art. If the fissure can be closed by surgical operation, that method is far preferable, for obvious reasons; but where that is impracticable, it only remains to undertake the adaptation of some artificial substitute.

From our own experiments, referred to in a former part of this account, we are satisfied that the most ingeniously contrived metallic velum will not be found to afford any material improvement to the articulation, though it may assist deglutition; because an instrument made entirely of gold or other metal, so as to accommodate itself to the movements of the surrounding soft parts, and thus close the fissure without producing irritation, would require a very delicate and complex structure, such as must always be liable to derangement from any of the shocks to which, from its position, it would be constantly exposed. If, in order to avoid this contingency, the instrument is made upon a more simple plan, and stronger, then it will not possess the necessary delicacy and flexibility. These obstacles have often suggested the expedient of attaching pieces of gum elastic to gold plates, which, occupying the same position as the natural velum, it was expected would perform its functions. We cannot learn that these oft-repeated plans have been brought to afford any permanent benefit, and, indeed, it seems to us they could not possibly succeed; for if the crude material employed is moulded to the shape required, its original form and consistency will soon be destroyed by exposure to moisture, animal heat, and muscular motion, all at the same time. Added to this, the surface of the gum soon becomes coated with a deposit of the farinaceous particles of the food, besides the tartar and other impurities, which must very soon render the continued use of the instrument highly objectionable.

Mr. Goodyeare's mode of preparing the caoutchouc enables us at once to get rid of all these objections, as the material he furnishes is found to retain the peculiar pliability of the original gum, and also the equally important quality of durability in resisting the combined action of the destructive chemical agents to which it must be subjected; and for that very reason, it has no affinity for extraneous substances by which the adhesion and accumulation of particles is promoted.

The writer has already given a history of the progress of the invention of the instrument through all the stages which have brought it to its present plan and form of construction. It was his original intention, at this point, to present a more detailed description of the mechanism; but we are aware that such descriptions are not read by one in one hundred, as they are, at the best, very obscure, since mere words, without the aid of figures or drawings, must always fail to convey a satisfactory idea of any purely mechanical contrivance. In this particular case we could not hope that diagrams representing objects bounded by irregular curves, and not by straight lines, circles, or geometrical figures, would afford the reader any clear notion of the affair; for the same reason that a student of anatomy would fail to acquire any practical knowledge of the shape and relative position of the parts of the animal mechanism from drawings and plates alone, without the advantage of demonstration and private dissection. But in lieu of description or drawings, the instrument itself may be seen, and the results of its application witnessed, by such as may have become interested in the preceding account. The processes employed in fabrication are also peculiar, and do not admit of description, but must be seen and practised in order to be understood.

From having been induced, by peculiar motives, to employ so much time upon this subject, more than any one else is likely to do, we are perhaps enabled to add some further hints which will be of use to others who may hereafter engage in a similar undertaken. Durability of the instrument being secured by the nature of the material employed in its construction, its efficiency depends upon its accurate adaptation to the shape of the surrounding parts. All mechanism of a complex nature must absolutely be avoided, that no prominent points or angles may be presented for the lodgment of food, or to obstruct its passage. It is necessary that it should possess the exact medium of flexibility, together with a perfect and permanent elasticity of substance. This will enable it to accommodate itself readily to the slightest muscular motion, so that it may be retained in place while eating, drinking, or even during sleep, without irritating the sensitive surface with which it is at all times in close contact. At the same time, it must possess firmness and consistency enough to sustain the force of sudden shocks, as in laughing, coughing or sneezing, without the possibility of injury or derangement. Lastly, it must readily free itself from all accumulations of food, deposits of tartar, and other impurities which might obstruct its regular action, or cause fœtor. To make the instrument still more perfect, an attempt has been made to give the velum a delicate pink color, resembling that of the natural flesh. Though that would, of course, add nothing to its practical value, yet a good deal of time has been spent to attain this last point without complete success.

The application of the instrument has been attended with the following circumstances:—From the yielding nature of the material employed, and the smooth surface given to it in the course of preparation, its first introduction would not be expected to cause any pain. At first, some inconvenience was of course experienced from introducing a strange piece of mechanism into a region peculiarly sensitive to the presence of foreign bodies. This occasioned a flow of saliva, continuing for a few days, after which, all inconvenience ceased. The first observable effect upon the speech was to give it decidedly more volume, and therefore render it more intelligible. Subsequent elocutionary practice was attended with the most encouraging results, and a marked improvement was perceptible from week to week. Exercise in the utterance of the simple vowel sounds on a low or base note, and endeavoring to produce a clear musical tone, was found sensibly to diminish the characteristic nasal sound. The articulation of each one of the consonant letters was at first practised separately, as children are taught penmanship, learning each letter by itself, and afterwards forming them into words and sentences. Pursuing this regular method through the whole alphabet, all the letters were at length perfectly acquired. D and G being semi-mutes (see Dr. Arnott's table) were the last and most difficult.

From circumstances already mentioned, it will readily be supposed, that when the writer first devoted himself to the task of supplying a mechanical remedy for congenital fissure of the palate, he did not contemplate pursuing the matter further than the particular case with which he commenced. But he was strongly advised, by one or two eminent surgeons who saw the invention, to prosecute it with a view to its general adaptation, and he has therefore with that design spent considerable time in making it as perfect as possible.* While occupied in that way, he has been surprised to find that the lesion is one of so frequent occurrence,† as the great proportion of hare-lip cases are complicated with fissure, and that a less proportion of the cases are found to admit of the application of a surgical remedy than was supposed. A coincidence of favorable circumstances, rather than any peculiar fitness or ability on his part, has contributed to a successful result. During his operations and experiments, he has arrived at principles of construction, and invented processes and methods to facilitate the business of fabrication, which, taken altogether, will be found to constitute a new and distinct art, and one which cannot be wholly imparted by the medium of a written treatise, but must be seen and practised in order to be understood. And this art, it is hoped, may be perpetuated as long as the necessity for it shall continue to exist.

* Dr. Mott, of New York, whose name is familiar to the profession in this country, was the first medical gentleman to whom I exhibited the invention, and the terms in which he expressed himself in relation to it led me to attach more importance, and devote more attention, to it than I might otherwise have done. A great deal of the substance of this account was originally contained in a paper which I prepared, at the request of Dr. Mott, for insertion in his late translation of M. Velpeau's Surgery, but by some mischance in communication, it failed to be received in season for the first volume, and I am now induced to publish it in this form.

† With regard to the comparative frequency of congenital fissure, I think, from what I can learn in conversation with eminent surgeons, that I am warranted in supposing that it occurs about as frequently as hare-lip, there being cases of fissure without hare-lip, and of hare-lip without fissure.

